# The Skin Microbiome: Current Techniques, Challenges, and Future Directions

**DOI:** 10.3390/microorganisms11051222

**Published:** 2023-05-06

**Authors:** Tasha M. Santiago-Rodriguez, Brice Le François, Jean M. Macklaim, Evgueni Doukhanine, Emily B. Hollister

**Affiliations:** 1 Diversigen, Inc., New Brighton, MN 55112, USA; jmacklaim@diversigen.com; 2 DNA Genotek, Kanata, ON K2V 1C2, Canada; brice.lefrancois@dnagenotek.com (B.L.F.); evgueni.doukhanine@dnagenotek.com (E.D.)

**Keywords:** culture-independent methods, metatranscriptomics, shotgun metagenomics, skin microbiome

## Abstract

Skin acts as a barrier that promotes the colonization of bacteria, fungi, archaea, and viruses whose membership and function may differ depending on the various specialized niches or micro-environments of the skin. The group of microorganisms inhabiting the skin, also known as the skin microbiome, offers protection against pathogens while actively interacting with the host’s immune system. Some members of the skin microbiome can also act as opportunistic pathogens. The skin microbiome is influenced by factors such as skin site, birth mode, genetics, environment, skin products, and skin conditions. The association(s) of the skin microbiome with health and disease has (have) been identified and characterized via culture-dependent and culture-independent methods. Culture-independent methods (such as high-throughput sequencing), in particular, have expanded our understanding of the skin microbiome’s role in maintaining health or promoting disease. However, the intrinsic challenges associated with the low microbial biomass and high host content of skin microbiome samples have hindered advancements in the field. In addition, the limitations of current collection and extraction methods and biases derived from sample preparation and analysis have significantly influenced the results and conclusions of many skin microbiome studies. Therefore, the present review discusses the technical challenges associated with the collection and processing of skin microbiome samples, the advantages and disadvantages of current sequencing approaches, and potential future areas of focus for the field.

## 1. Introduction

Acting as the largest organ of the human body, the skin serves various functions. For instance, skin acts as a sensory organ, regulates temperature and moisture, provides a physical barrier against thermal and physical damage, and protects against invading pathogens [1,2]. The aforementioned functions are primarily driven by the structure, physiology, and biology of the skin. Skin is composed of two layers, namely, the dermis and epidermis, which are characterized by diverse, specialized niches or micro-environments that vary with respect to pH, ultraviolet (UV) radiation exposure, oxygen availability, temperature, moisture, and/or sebum content [1]. The location and distribution of sweat, sebaceous, and apocrine glands across the skin are well known to have a significant influence on these micro-environments [1]. Differences in the attributes of the skin surface, such as those mentioned above, are known to influence the composition and distribution of the skin’s resident microbes, collectively known as the skin microbiome, as well as their ability to maintain healthy conditions or promote disease [3,4].

A global and interdisciplinary project was developed to understand the microbial components of our genetic and metabolic landscape and their contribution to human physiology and disease predisposition. Also known as the human microbiome project (HMP), which launched in 2007 [5], it demonstrated that healthy human skin is dominated by one of three main genera (*Cutibacterium* (formerly *Propionibacterium*), *Staphylococcus*, and *Corynebacteria*), with community composition and diversity varying significantly between subjects and across sites [6]. Follow-up work on a larger cohort showed that the biogeography of each skin site is the primary determinant of the taxonomy and functional characteristics of its resident community [7,8]. These studies also highlighted the individualized nature of the skin microbiome, with each donor harboring unique microbial signatures and bacterial strains. The skin microbiome, which is composed of bacteria, viruses, archaea, and fungi, is associated with maintaining a healthy status and interacts with the immune system in the process [9]. While it is estimated that from 10^4^ to 10^6^ bacteria inhabit each square centimeter of skin [10], new microbial species continue to be discovered whose distribution and function depend on the various specialized niches of the skin [11]. For instance, oily skin sites are highly acidic and are characterized by bacteria that can consume lipids and require or may survive under anaerobic conditions (e.g., *Corynebacterium minutissimum* and *Cutibacterium* spp.) [2,12,13]; moist skin sites, which are mildly acidic environments with higher temperatures and humidity contents, are home to bacteria known to contribute to body odors (e.g., *Corynebacterium* spp. and *Staphylococcus* spp.) [2,12]; and dry skin sites are known to have the lowest biomass and yet the greatest bacterial diversity relative to other skin sites [2,12]. While bacterial profiles have been more extensively characterized, fungi and viruses are also common components of the skin microbiome. For instance, *Malassezia* spp., including *M. restricta*, *M. globose*, and *M. sympodialis* are known to be the main skin fungal commensals, or mycobiome [2], and their prevalence and abundance can differ based on the micro-environments of the skin [14]. The viral fraction, or virome, can be highly individualized; bacteriophages are some of the major components, particularly those that infect prevalent members of the skin bacterial community (e.g., *Cutibacterium* spp., *Corynebacterium* spp., and *Staphylococcus* spp.) [6]. Eukaryotic viruses, such as those belonging to the *Papillomaviridae* and *Poxviridae* families, have also been identified in skin samples, and their presence tends to be highly personalized [6]. Notably, mycobiome and virome research lags significantly behind bacterial microbiome studies mainly due to their lower relative abundance on skin compared to bacteria and the technical challenges associated with their extraction and characterization [14].

The skin microbiome is seeded at birth and is influenced by factors such as delivery mode, the mother’s microbiota, antibiotic treatment, hygiene, nutrient deficiency, housing, animal/pet contact, and environmental exposure [15]. Microbial colonization at birth and associated factors are known to be essential for immune system development [16]. Factors such as host physiology, biological sex, age, skin site, climate, geographical region, lifestyle (e.g., occupation and hygiene), immune system, host genotype, and underlying conditions (e.g., diabetes) can also affect the skin microbiome throughout life [1] and have been reviewed in detail elsewhere [17]. It is also known that certain physiological characteristics of the skin, including sebaceous gland function, blood flow, and pH, may be intrinsically associated with changes in the diversity and composition of the skin microbiome. For instance, a study found that skin bacterial diversity decreases with age, regardless of skin site, and that the relative abundances of specific bacterial genera such as *Lactobacillus* and *Cutibacterium* decrease on aging skin [18]. Similar outcomes have been demonstrated in subsequent studies, showing that the skin microbiome associated with younger women (19–28 years old) is significantly different from the skin microbiome of older women (60–63 years old) [19]. Aging skin is also known to affect pathogen colonization and may delay wound healing, particularly in conjunction with conditions such as diabetes, or as the immune system becomes senescent [20,21]. Interestingly, another study found that chronological age alone does not correlate with the bacteria on the skin [22]. These studies highlight that the potential determinants of skin health and disease are difficult to tease out and are just beginning to be elucidated.

Specific associations between the skin’s physiology and its microbiome, as well as specific microbe–microbe and microbe–host competitive and synergistic interactions, have been identified and elucidated in part by culture and culture-independent methods [2]. However, not all skin microbes are necessarily recoverable via culture. This can lead to an underestimation of microbial richness, making it a significant source of bias. Culture-independent methods include amplicon and/or untargeted short and long read sequencing and may circumvent some of the limitations of culture-dependent methods. While both sequencing approaches are subject to challenges associated with skin’s typically low levels of microbial biomass (hereafter referred to as low biomass), amplicon-based approaches have been widely adopted and benchmarked to overcome the high host content present in skin samples. Skin is also an externally facing environment; thus, it may be highly influenced by the environment and prone to contamination/bioburden [23].

**Figure 1:**
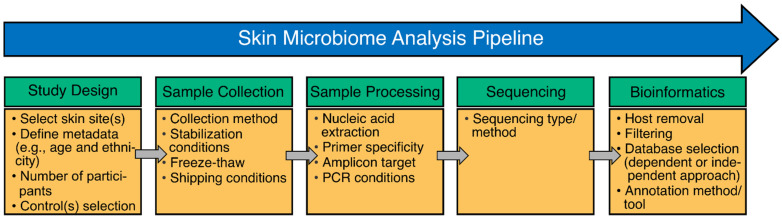
Overview of a standard skin microbiome analysis pipeline. A skin microbiome analysis pipeline includes study design, sample collection, processing, sequencing, and bioinformatic analysis. Some steps within sample processing and bioinformatics may be specific to certain sequencing approaches (e.g., amplicon target within sample processing is specific for targeted or amplicon sequencing and host removal may be specific for untargeted sequencing). The figure also shows several of the steps that may add biases to a skin microbiome pipeline (discussed in this review); these were also previously described for microbiome studies and applied in skin microbiome research [24]. PCR: polymerase chain reaction.

Regardless of the sequencing approach, the overall process of studying the skin microbiome is similar in that samples are collected, nucleic acids are extracted and sequenced, and data are analyzed (Figure 1). Notably, skin microbiome research is not without limitations as several of the previously mentioned steps have significant potential to introduce bias in results and interpretation. This will be described in greater detail in this review. Thus, the present review focuses on the technical challenges associated with the study of the skin microbiome (from study design to sequence data analysis), the advantages and disadvantages of current sequencing approaches, and finally discusses potential areas of development to help advance the field with respect to areas of discovery and therapeutic applications.

## 2. Methods to Study the Skin Microbiome and Associated Biases

Historically, members of the skin microbiota have been studied using culture-based approaches in which cells collected from a site of interest are grown in appropriate media with the purpose of identifying a specific species/pathogen. The culturing of skin microbes, particularly bacteria, is important for antibiotic susceptibility and virulence testing, as well as genomic and proteomic analyses [25]; however, not all skin microbes are necessarily recoverable via culture, which can lead to significant underestimation of microbial richness, making it a significant source of bias. Molecular-based approaches, such as amplicon and/or untargeted sequencing (e.g., short- and long-read sequencing, discussed in this review) may help circumvent the limitations of culture-based methods [25]. However, the study of the skin microbiome using sequencing approaches is not without its limitations, which mainly stem from the skin’s intrinsic low-biomass nature, high bioburden, and the relatively high abundance of host DNA across most sites. The following section describes the main technical challenges, biases, and considerations associated with each step of a skin microbiome study using sequencing approaches. These considerations are also summarized in Table 1. While some steps focus on amplicon sequencing, several others, including study design, sample collection, database selection, and analysis, also apply to all sequencing technology.

### 2.1. Study Design

Regardless of the sequencing approach (i.e., targeted amplicon vs. untargeted shotgun), study design is the first step for a successful study [26], and it is incredibly important for skin microbiome research given the numerous sources of biases introduced at the various steps of the workflow (Table 1). Several factors must be considered when designing a skin microbiome study. These may include, but are not limited to, understanding the ecology of the skin site(s) of interest, such as its (their) overall biomass and host content, the impact of disease/skin condition (e.g., inflammation), participants’ characteristics (e.g., hygiene, metadata, and health status), the number of participants needed to answer a research question, sample replication or longitudinal (repeated within individual) sampling, optimized collection method, storage, extraction, and analyses tailored to the study (Figure 1). The interpretability of the results could be impacted by not considering all the above-mentioned factors in a skin microbiome study. The inclusion of control(s) in several or all of the above-mentioned steps is also critical for assessing sample performance and reliability throughout the study in its entirety [26].

### 2.2. Sample Collection and Storage

After study design, sample collection and storage are crucial steps in a skin microbiome study design (Figure 1). Improper sample handling can impact the ability to preserve and detect the true microbial profiles and, in turn, affect the understanding and/or interpretation of microbial diversity, patterns, and dynamics (Table 1). Skin microbiome samples are typically collected using a swab, sticky tape, scraping, or, alternatively, a biopsy from the site of interest. Each method has pros and cons, with swabs generally thought to be better suited to collecting the “surface” microbiome, while sticky tape and/or scraping can presumably recover microbial cells present in the deeper layers of the epidermis (i.e., the microbiome found in pores and glands) [25]. In contrast, biopsy samples can capture a greater cross section of the skin ecosystem since the dermis is also sampled and may subsequently provide differing results that may be more informative for dermal skin diseases and conditions [25]. While early and recent studies have shown that certain taxa can be captured by both swab and biopsy collection methods, each method can also capture unique taxa [27,28]. Similarly, while tape and swab collection methods can show similar results in terms of DNA community profiles, alpha diversity, and clustering patterns, the tape collection method seems to select for the recovery of aerobic bacteria [25]. Notably, swab collection methods have the advantage of being widely commercially available, standardized, and, in some cases, validated; as such, they enable greater consistency in results across various skin sites and studies [29]. Recently, collection techniques that penetrate deeper within the epidermis and epidermal invaginations, such as the microprojection array, have showcased their effectiveness in capturing a more complete representation of the skin microbiome, particularly in recovering higher fungal diversity, such as the Ascomycota phylum [30]. A combinatorial strategy for skin microbiome collection using multiple devices may be warranted to ascertain the full complement of taxonomic diversity while also keeping in mind donor compliance and cohort recruitment challenges for the more invasive collection methods.

In order to prevent changes to sample integrity and community composition post-collection, skin microbiome samples are collected in either the laboratory or the clinic and immediately stored at −80 °C, or they are collected remotely and returned in a tube that contains a stabilizer that can maintain microbial nucleic acid quality and the microbial profiles and relative abundances during storage and/or return shipping [31]. Various preservation approaches, in combination with collection methods, have been tested and demonstrated to impact profiles within specific skin sample sites [32]. Skin microbiome studies should rely on a collection strategy with the ability to capture and preserve the known biological community of the skin site of interest which will also mitigate the introduction of contaminating DNA or RNA (i.e., bioburden) as much as possible. Given the extremely low biomass nature of skin, the collection solution should also facilitate the maximum recovery of nucleic acids in downstream processing steps (Table 1).

### 2.3. Sample Processing: Nucleic Acid Extraction

The recovery of DNA from a collected sample is known to be one of the critical steps of sample processing, and it can introduce variation or contribute to biases towards specific bacterial groups (in particular, the over-representation of Gram-negative bacteria) in skin microbiome studies (Figure 1 and Table 1) [33]. Typical extraction methods leverage enzymatic (e.g., proteinase K and lysozyme) or mechanical lysis to ensure the recovery of both Gram-positive and Gram-negative bacterial species. Sub-optimal sample extraction can lead to significant bias in the samples (i.e., the overrepresentation of easy-to-lyse bacteria) [34]. Different nucleic acid extraction protocols can have high variability in their extraction efficiencies, further biasing results and convoluting comparative results across studies relying on different extraction methods. Given the low microbial load of skin, extraction should minimize the introduction of an external bioburden (such as the “kitome”, discussed in this review) that could confound interpretation of the sample’s true microbiome [33]. For instance, a study addressing the DNA extraction success rate from 12 different commercially available kits found that DNA concentrations and successful sequencing library preparation can vary drastically depending on the commercial extraction kit used [33]. The effective recovery of fungal nucleic acids is highly dependent on the extraction workflow, as showcased for skin and other sample types when comparing commercially available microbiome extraction solutions [35]. In the case of tape-stripping and swabs, it is important to ensure that the collected material is released from the tape/swab prior to DNA extraction to avoid potentially under-sampling the DNA and the misrepresentation of the microbial content. The release of cells from tape is generally harder than from swabs, resulting in relatively low levels of “extractable DNA”, which can greatly impact sample performance in downstream sequencing assays (especially for shotgun metagenomic sequencing, which generally requires higher input compared to targeted amplicon sequencing). Although, many insights have been generated on the skin microbiome in the recent past, the general lack of standardization of the field (from sample collection to extraction) has likely impeded our understanding. Efforts should be made to develop standardized and validated tools and technologies specifically tailored to the study of low biomass samples such as skin.

### 2.4. Sample Processing: Amplification and Library Preparation

Pioneering (and ongoing) skin microbiome studies have relied on the amplification of specific regions of the 16S ribosomal RNA (rRNA) gene and internal transcribed spacer (ITS) to understand the skin’s bacterial and fungal community structures, respectively [3,36]. Both 16S rRNA gene and ITS amplicon sequencing continue to be the preferred methods in skin microbiome studies due to their relatively low cost compared to other sequencing technologies. In addition, targeted microbial amplification can avoid sequencing abundant host DNA, which often hampers the recovery of microbial sequence data in sequencing-based approaches. Early studies showed the presence of several bacterial phyla, with Actinomycetota (previously known as Actinobacteria), Bacillota (previously known as Firmicutes), Pseudomonadota (previously known as Proteobacteria), and Bacteroidota (previously known as Bacteroidetes) being the most predominant on skin [3]. As sequencing technologies, databases, and annotation methods became more robust, more specific taxonomic levels have been achieved, revealing that the skin is composed of species belonging, for the most part, to the *Staphylococcus*, *Cutibacterium*, and *Corynebacterium* genera [4]. Additionally, 16S rRNA gene amplicon sequencing has enabled the deciphering of the skin microbiome in relation to skin conditions. For instance, a study using the amplification of the 16S rRNA gene V1-V3 regions found that *Lactococcus*, *Porphyromonas*, *Prevotella*, *Neisseria*, *Acinetobacter*, and *Fusobacterium* are associated with psoriasis, although they are rarely identified on healthy skin [37]. Thus, it has been suggested that psoriatic patients may be more susceptible to colonization by environmental bacteria [37]. In other conditions such as atopic dermatitis (AD), sequencing of the 16S rRNA gene V1-V3 regions of skin samples showed that *Staphylococcus aureus* dominates the sampled lesion sites and correlates with disease severity [38]. These results show that amplicon sequencing has been and continues to be insightful in skin microbiome studies.

Amplicon sequencing is not without limitations, particularly during PCR amplification. For instance, primers targeting ITS regions 1 and 2 (ITS1 and ITS2) have been widely used in microbiome studies to understand fungal composition and structure [39] (Figure 1). However, it is still unclear which ITS fragment is optimal for fungal characterization in various sample types since most ITS primers fail to accurately profile mock communities [39,40]. This has resulted in inconsistent results, suggesting that biases in primer selection and amplification may affect the interpretation of results. Similarly, the interpretation of bacterial community structure can be biased as a result of the 16S rRNA gene primers used. While the 16S rRNA gene V4 region has been widely utilized in gut and environmental microbiome studies, skin microbiome research has benefited from amplification of the V1-V3 region of the 16S rRNA gene, which is currently considered the gold-standard [41]. One of the main reasons for the recommendation of the 16S rRNA gene V1-V3 primers in skin microbiome studies is that primers targeting the V4 region have failed to adequately recover *Cutibacterium acnes*, a key member of the skin microbiota, and have thus not been extensively used in skin microbiome studies [41]. However, a recent study using human skin microbiome samples demonstrated that the use of modified primers to modulate primer editing by high-fidelity DNA polymerases, such as KAPA, can minimize the dropout of taxa with mismatches to amplification primers. Editing primers targeting the 16S rRNA gene V4 regions results in the recovery of similar levels of *Cutibacterium* as those observed with 16S rRNA gene V1-V3 primers [42]. In addition to primer editing, there are several best practices that can decrease biases associated with PCR amplification which also apply to skin microbiome studies including, but not limited to, optimizing DNA input concentration [43], minimizing the number of PCR cycles to avoid false positives/false negatives and PCR saturation artifacts [44], and the use of high-fidelity polymerases to reduce errors in sequence amplification [45] (Table 1). Although amplicon sequencing is the current gold standard for skin microbiome research, it has thus far mainly helped researchers understand the composition of the skin microbiome across various skin sites/micro-environments. Accumulating evidence suggests that select skin conditions are not mediated by changes in the community structure or relative abundance of skin commensals but are driven by pathogenic strains of skin commensals [46]. As such, sequencing technologies with improved resolution are needed to better understand the role of the skin microbiome in health and disease.

### 2.5. Bioinformatics: Database Selection and Annotation

A critical step in the analysis of sequence-based data, either amplicon or shotgun metagenomic sequencing, is the choice of a reference database for assigning or annotating sequenced reads with taxonomic and/or functional information (Figure 1) [47]. Reference databases should be suited to the target of interest (e.g., in the case of targeted amplicon), or be comprehensive in genomic content representative of the genetic diversity of the expected skin microbiome. There are several considerations in the choice of reference database or for curating a custom solution. These include the content source(s), data quality (e.g., full-length amplicon or full genomic characterization), degree of curation (e.g., removing and/or consolidating ambiguous content or redundant sequences), the richness of the data annotation associated with the references, the recency of the data, and the comprehensiveness of skin-specific references covering a range of taxonomic origins, body sites, and cohort sources (Table 1).

Database selection should also match the analysis strategy and be suited for the sample type and context and the mapping or alignment tool of choice and provide robust and informative results (Table 1). Evaluating mock community controls, either as input for sample processing or as in silico communities of a known composition, can be a useful strategy for assessing annotation accuracy and for the ability to resolve closely related sequences to expected taxonomic levels. While the SILVA reference database dominates as an annotation source for 16S rRNA gene amplicon sequencing [48], there have been no comprehensive, benchmarked databases and analysis strategies for the shotgun metagenomic sequencing of the skin microbiome to date. The result is an abundance of choice and divergent methods in the field, creating study-specific biases in annotation results and interpretation which can potentially hinder the study-to-study comparability of results [49].

In general, amplicon-based sequencing (e.g., targeting a region of the 16S rRNA or ITS genes) provides less sequence variability compared to full genomic content for the ability to resolve species and sub-species level assignment, thus resulting in genus-level or higher taxonomic assignments [50]. Most recently, de-noising or amplicon sequence variant (ASV)-based methods of analysis have provided better sensitivity to identify true sequence differences and an improved ability to identify contaminants or spurious sequences that are non-informative (Table 1) [51]. These database-independent approaches allow investigators to identify single-nucleotide changes that co-occur with samples or study design without relying on reference assignment, potentially providing novel associations. Notably, ASVs are often classified to the nearest taxonomic match to bridge the clinical and therapeutic applications and interpretations.

## 3. Ongoing and Proposed Approaches to Study the Skin Microbiome

While amplicon sequencing has been widely benchmarked and validated for the study of the bacterial and fungal taxa found on skin, other approaches, including shotgun metagenomics, whole 16S rRNA gene amplicon sequencing, and metatranscriptomics, have also been tested or proposed as part of the toolbox of methods for the characterization of the community composition and function of the skin microbiome. These techniques are discussed in the sections below.

### 3.1. Shotgun Metagenomics

Shotgun metagenomics enables the sequencing of all DNA in a sample and can be used to understand bacterial, viral, and fungal community compositions at more refined taxonomic levels (i.e., species- and strain-level). It can also create opportunities for gene and functional analyses, the assembly of genomes or genomic fragments, strain tracking, and de novo discovery. Notably, fewer shotgun metagenomic studies of the skin microbiome have been performed compared to amplicon studies. This mainly stems from the higher costs associated with shotgun metagenomic sequencing and the relatively high proportion of host DNA in skin samples, which warrants a higher sequencing depth. Several in vitro host DNA depletion strategies have been developed and tested, but they often require fresh samples and have limited efficacy/high potential for biasing the microbial DNA composition [52]. Beyond these limitations, shotgun metagenomic sequencing is better able to capture the taxonomic accuracy and diversity of skin microbiome communities, and unlike amplicon and near-full-length 16S rRNA gene sequencing, can improve strain-level identification while providing insights into the microbial function present in the community [41,53].

Shotgun metagenomic sequencing has enabled important findings regarding the skin microbiota. For instance, one study found that facial sites, including the cheeks, forehead, and back or inside of the nose, are characterized by distinct bacterial species compositions and functions [54]. Specifically, porphyrin-producing *Cutibacterium* species, such as *C. acnes*, *C. avidum*, *C. granulosum*, and *C. namnetense*, are more prevalent on the back of the nose compared to other facial sites [54]. While porphyrins are important metabolites for the synthesis of essential molecules, they are also known to be pro-inflammatory, and higher porphyrin levels have been associated with skin conditions such as acne [55]. Another study that applied ultra-deep shotgun metagenomic sequencing showed higher relative abundances of Propionibacteria (more recently classified as *Cutibacterium*) phages on the skin of healthy individuals compared to a greater diversity of *C. acnes* strains in subjects with acne, potentially revealing a modulating effect of the bacteriophage on bacterial composition. In addition, virulence factors were enriched in subjects with acne [56], further demonstrating that the information recovered from shotgun metagenomic sequencing extends beyond bacterial taxonomic identity. This study also demonstrates the need for appropriate and cost-effective sequencing solutions for skin microbiome studies that would enable strain identification. Shotgun metagenomics sequencing is highly attractive for the study of the human skin microbiome given its ability to improve taxonomic resolution while providing insights into functional potential. However, the high level of host DNA has been a major hurdle to its widespread adoption. Other approaches, such as multi-locus strain typing (MLST), can be readily applied to shotgun metagenomic sequencing datasets to enable strain typing at relatively low depths, and are valuable for high-host-content samples such as skin [57,58].

Recently, shotgun metagenomic sequencing approaches for the improvement of taxonomic recovery that were first developed for gut microbiome studies have been tested and translated to a skin microbiome use case. In particular, a hybrid method of typical short-read metagenomic sequencing combined with long-read sequencing (e.g., PacBio or Nanopore technologies), discussed in this review, has been identified as a means of combining the unique biodiversity recovered by the individual methods [59]. A study comparing short- and long-read sequencing and a hybrid approach across arm and foot skin microbiomes highlighted the improvement in metagenomic assembly and coverage with the hybrid approach, particularly for low-abundance species, demonstrated by the discovery of *Corynebacterium simulans*, a previously uncharacterized species, and for evaluating strain heterozygosity [60]. While such techniques are still novel and require a combination of sequencing platforms, separate library preparations, and informatics techniques that can effectively utilize data of different read lengths, the benefits in sensitivity can be crucial in teasing out the complexity of skin microbiomes [59].

### 3.2. Whole 16S rRNA Gene Sequencing

Although the 16S rRNA gene has a long history of use as a marker gene for bacterial phylogenic assessment and taxonomic identification [61,62,63], the advent of short-read high-throughput sequencing technologies necessitated the use of shorter fragments as proxies for the full gene sequence information due to the read-length limits. As a result, most current skin bacterial community composition analyses are based on the amplification and sequencing of specific variable regions of the 16S rRNA gene. However, the amplification of a limited number of variable regions of the 16S rRNA gene may hamper interpretation and optimal taxonomic resolution because of amplification biases and the limited sequence variability in shorter fragments [64]. For this reason, sequencing the whole 16S rRNA gene can help overcome biases associated with analyses of shorter regions and may improve taxonomic resolution, enabling species- and strain-level differentiation [64]. Early skin microbiome studies leveraged near-full length 16S rRNA gene sequencing and analysis with Sanger sequencing, and this approach continues to be used in diagnostic settings [65]. Multiple primer sets are used in Sanger sequencing which generate overlapping amplicons that can be built into contigs that may cover most of the 16S rRNA gene. However, Sanger sequencing is low-throughput, more laborious, and less cost-effective than next-generation sequencing technologies.

For these reasons, third generation long-read sequencing technologies have gained popularity for obtaining species- and strain-level resolution in microbiome studies by sequencing complete or near-complete 16S rRNA gene sequences in a single long read. One caveat of such technology is the higher sequencing error rates associated with long-read technologies, which require specific data processing to account for and correct [66]. Advances in synthetic long-read sequencing, such as those by LoopSeq for the full-length 16S rRNA gene, have made strides in overcoming some of these limitations [67]. In addition, the comparatively low throughput of these technologies and the higher cost per sample are limiting their wider adoption in population-wide microbiome research studies and rapid diagnostic device development. However, long-read sequencing technologies have been applied to characterize the species and strains of the skin microbiota in research settings. A study applying third-generation sequencing on mock communities composed of known skin bacteria and skin samples showed that all reads covering the V1-V9 regions of the 16S rRNA gene were classified at the species level [68]. However, this study also showed an over-representation of *Staphylococcus* and under-representations of *Cutibacterium* and *Corynebacterium* [68]. This suggests that while species- and strain-level resolution are achieved with near-full-length 16S rRNA gene sequencing, the technique may not necessarily capture the relative composition of skin samples accurately because of primer bias. Rather, the technology may be used to augment information acquired from short-read sequencing technologies to understand species- and strain-level variation, particularly in cases of therapeutic or diagnostic applications, which may require a high degree of taxonomic resolution.

Several areas of improvement for near-full-length 16S rRNA gene sequencing are on the horizon as the technology matures, such as the selection of appropriate amplification enzymes to reduce taxonomic bias due to guanine and cytosine (GC) content variation [68,69], the use of degenerate primers to overcome sequence heterogeneity across species and strains [70], and the inclusion of ITS and partial 23S rRNA gene during the amplification process. The latter has been successfully employed, with evidence for generating substantially higher unique taxa across samples and attaining significant strain-level resolution when compared to metagenomic sequencing [68]. As with 16S rRNA or ITS gene amplicon sequencing, near-full-length 16S rRNA gene sequencing does not provide information about other life domains or function; thus, the application of techniques such as shotgun metagenomics and metatranscriptomics can provide gene and functional information in addition to taxonomic annotation.

### 3.3. Metatranscriptomics

In contrast to shotgun metagenomic sequencing, metatranscriptomics is the study of the gene expression of a community as a whole and provides insights into the transcriptional activity and response of a community to a temporal context [71]. To capture metatranscriptomic sequencing information, the sample workflow typically includes total RNA isolation, rRNA depletion, the reverse transcription of messenger RNA (mRNA) to complementary DNA (cDNA), and cDNA sequencing. Bacterial rRNA depletion in complex samples can be challenging [72], and insufficient depletion leads to a high proportion of rRNA reads, which does not provide information on the functional activity of the microbiome. Other challenges include the adequate recovery of RNA from low-biomass skin samples and the potential for a high level of host RNA that effectively diminishes the recovery of microbial mRNA signals, requiring higher sequencing depths to overcome these obstacles [73]. Different sources of functional information can be used to assign annotation to reads, including, for example, Pfam [74], the Kyoto Encyclopedia of Genes and Genomes (KEGG) [75], Clusters of Orthologous Genes (COG) [76], and Swiss-Prot [77]. As with metagenomic annotation, the choice of reference and annotation tool may be critical for the interpretation of results. In contrast to metagenomic sequencing, taxonomic profiles generated from metatranscriptomic data can represent the functional activity of discrete taxonomic groups in which fractions of the community may be alive and functionally active, while other taxa are present but not necessarily alive, metabolically active, or responsive to the environment [71]. This second point is particularly noteworthy in the context of the skin microbiome given the degree of environmental exposure the skin is subject to and the potential for skin to carry dead microbes and an environmentally derived bioburden in addition to live microbes, which are typically difficult to distinguish from one another using DNA-based methods [23,78].

Within the skin microbiome field, investigators have used metatranscriptomic sequencing to follow the dynamics of microbial communities in diabetes-related foot ulcers in response to debridement and the application of a topical surfactant gel [79] and in burn wound infection clearance [80], as well as to demonstrate that the transcriptional activity of skin microbes differs between acne patients and controls and that vitamin B_12_ supplementation can modulate the transcriptional activity of skin microbes such as *C. acnes* [81]. To date, however, the use of metatranscriptomics in the study of the skin microbiome has remained limited to a small number of studies, and it is often limited to targeted measurements of gene expression (e.g., using Reverse Transcription (RT)-quantitative PCR (qPCR)). As a technique, metatranscriptomics is challenging given the short half-life of bacterial mRNAs; as such, it requires the rapid collection and effective stabilization of samples prior to nucleic acid extraction [73]. As new and improved techniques and technologies emerge and address the challenges associated with sampling, capturing, preserving, and recovering high-quality RNA from skin and effective rRNA and host depletion, it is anticipated that skin metatranscriptomic studies will become more frequent and provide valuable insights into the taxonomy, functional outputs, and changes in the dynamics of the skin microbiome and its interplay with host physiology.

## 4. Assessing Reagent and Cross-Contamination in Skin Microbiome Studies Using Controls

Skin microbiome samples are susceptible to the negative effects of contamination due to the low (microbial) biomass nature of most skin surfaces. The contamination of skin microbiome samples can originate from multiple sources, including laboratory reagents (i.e., the kitome) and/or samples that are mishandled during collection, extraction, amplification, library preparation, and sequencing, all of which may result in sample contamination [82]. Contamination by reagents and cross-contamination have the potential to hamper the identification of relevant biological signals in skin microbiome samples. Contamination risks in skin microbiome studies can be assessed and potentially mitigated by including and sequencing controls. There are different types of controls that may be included to help identify the kitome and cross-contamination and to assay performance. These include, but are not limited to, extraction blanks, no-template controls for library preparation, positive controls, and mock communities (Table 2). The failure to include, sequence, and analyze such controls may impact the interpretation of the results, undermining the study’s robustness. This is particularly important due to the propensity of sequencing platforms to capture contaminating sequences even when there is a lack of nucleic acid detection by upstream quality control (QC) metrics and positive product amplification. Several of the above-mentioned controls may also serve the purpose of addressing extraction and amplification biases, sequencing efficiency, and accuracy in data annotation and analysis (Table 2). For instance, while in silico mock communities cannot address biases associated with steps upstream of data analysis, these are usually included as a control to assess the number of reads mapping to a particular database, as well as the accuracy of the annotation tools used.

“Prevention is the best cure”, and several best practices can be applied to minimize the impact of reagent contamination and cross-contamination on skin microbiome results when adding one or several of the above-mentioned controls. For instance, including multiple controls that address the above-mentioned issues and randomizing samples and controls when extracting nucleic acids and when preparing and sequencing libraries can help minimize and/or aid in addressing contamination issues. It is noteworthy that although there are strategies to remove contaminating sequences bioinformatically, in practice, this is a complex approach, and some types of contaminants (e.g., sample-to-sample contamination) are exceedingly difficult to identify and remove without impacting confidence in the resulting sample profile. Moreover, skin is a common contaminant itself since it is easily introduced by the operator; as a result, this practice is not broadly accepted since some of these sequences may also be part of the authentic microbiota of interest. However, there are tools that have been developed to identify and remove contaminants originating from reagents. Some of these tools include, but are not limited to, SourceTracker [83] and Meta-SourceTracker [84] to predict sources of contamination from amplicon and shotgun metagenomics data, respectively, as well as decontam (amplicon and shotgun metagenomics data) [85], microDecon (amplicon data) [86], Recentrifuge (shotgun metagenomic data) [87], and Squeegee (shotgun metagenomics data with the possibility of application to amplicon data) [88]. Notably, these tools often require experimental designs with multiple controls to support contaminant removal and still need to be extensively tested in skin microbiome datasets suspected to be contaminated with external sources of microbial DNA.

## 5. Future Directions and Applications of Skin Microbiome Research

**Figure 2:**
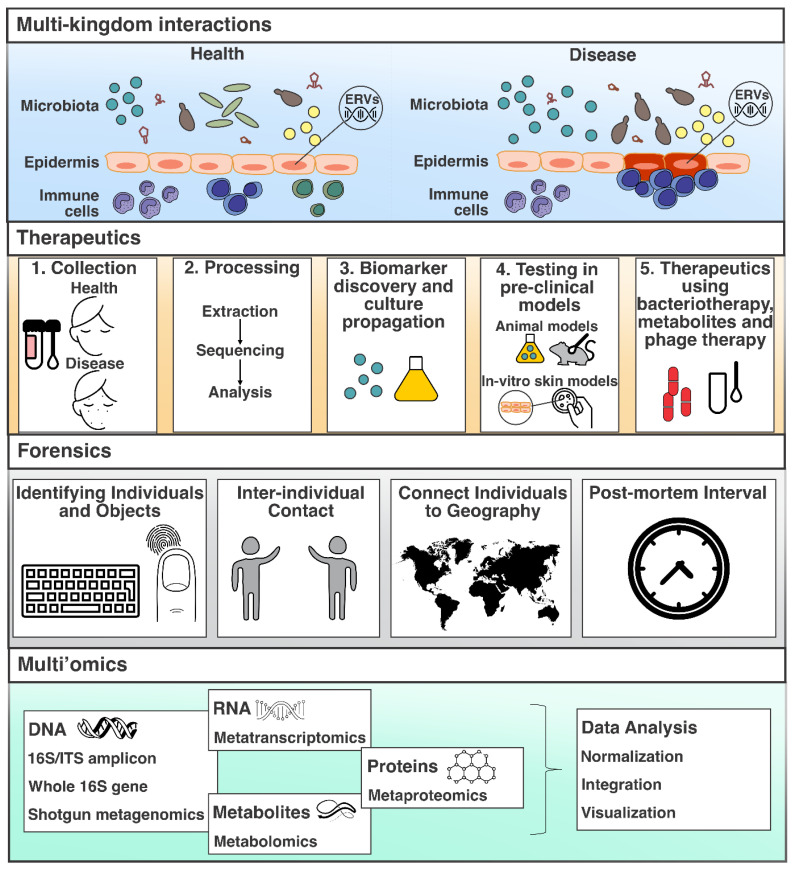
Highlights of some of the potential future directions and applications of skin microbiome research. These include, but are not limited to, deciphering multi-kingdom interactions, developing therapeutics, forensic applications, and integrating various ‘omes from skin microbiome studies, also known as multi’omics. ERVs: endogenous retroviruses.

As laboratory and bioinformatic techniques and technologies continue to be developed and validated for skin microbiome research and additional approaches are developed in the field (e.g., metatranscriptomics), novel insights into the skin microbiome will soon be generated. For instance, multi-kingdom interactions can be unraveled, novel therapeutics can be developed to treat dermatological conditions, forensic applications can be established, and the integration of datasets originating from multiple technologies (multi’omics) can be considered (Figure 2). These aspects are discussed in the section below.

### 5.1. Unraveling Multi-Kingdom Interactions

Members of the skin microbiota are known to interact at molecular and immunological levels with organisms of other life domains, processes also known as multi-kingdom interactions (Figure 2). On skin, these multi-kingdom interactions can occur between and across bacteria, viruses, archaea, fungi, and the host at specific taxonomic levels (i.e., species, subspecies, and strain) [89]. Species-, subspecies-, and strain-level diversity can often be subject-specific [89], possibly suggesting that certain multi-kingdom interactions may also be subject- and disease-specific. For instance, subjects with atopic dermatitis (AD) have shown higher *Staphylococcus epidermidis* strain diversity across cohorts, creating the opportunity to elucidate strain-level variation in association with AD flares [90]. Interestingly, this strain diversity on the skin has also been associated with specific functional activity in which certain *S. epidermidis* strains are known to produce damaging proteases [91]. In addition, the capacity of skin bacteria to interact with the host and trigger immune responses in association with AD has been determined by challenging human keratinocytes with live bacteria, including *S. aureus* and *S. epidermidis* [90]. These results support specific bacteria–host interactions in association with health and specific dermal disease phenotypes. Another study showed that the intensity of immune responses to the microbiota in both health and in association with inflammation are controlled by the expression of endogenous retroviruses (ERVs) [92], which comprise a substantial part of human and animal genomes [93]. The study found that the expression of ERVs may be more discrete in a healthy state but higher during inflammation. This study also showed that skin health and disease may be a function of these viral interactions with the immune system and that the host may have ultimately selected its endogenous virome to communicate with the exogenous microbiota. These results also create the opportunity to further understand the potential role(s) and interactions across other life domains (e.g., viruses and fungi) with the host and its immune system in relation to skin health and disease.

### 5.2. Developing Therapeutics and Diagnostics

Developing therapeutics for dermal diseases is one of the long-term goals of skin microbiome research, and sequencing information is increasingly being used for such purposes (Figure 2). Ideally, developing therapeutics requires a case–control study design with the collection of samples from both healthy controls and patients at skin sites showing signs of the disease of interest [2]. Bacterial or any other microbial components of interest should then be tested in ex vivo, in vivo, or in vitro pre-clinical models to determine potential effects. If the microorganism of interest can capture features of the disease in animal models and/or skin systems, additional experiments can then be performed to explore the disease mechanism(s). A therapeutic can then be developed and further tested, depending on the results from the previously mentioned steps. A similar described model could be used to identify beneficial microorganisms that are present in controls but lacking in patients and that potentially could be used as probiotics for bacteriotherapy [94]. In skin bacteriotherapy, the ecology of skin with a specific condition can be restructured using a bacterial consortium with beneficial properties as a form of skin microbiome transplantation [95]. Alternatively, skin bacteriotherapy may also work by, for example, targeting a specific pathogen associated with a dermal condition by using specific metabolites produced by specific bacteria [94] or through the application of phage therapy. In particular, the use of bacteriophages as a topical therapy has proven to be effective in targeting *C. acnes* and ameliorating acne [96]. Phage therapy, however, also comes with its own challenges, particularly those associated with direct and indirect immune responses and arms races, contributing to bacterial and viral evolution, which can ultimately influence the efficiency of phage therapy in skin restoration approaches [96,97,98].

In addition to developing therapeutics, diagnostics based on skin microbiota may provide more precise treatment for conditions such as acne, rosacea, psoriasis, and atopic dermatitis [99]. Beyond the study of the most common skin environments (i.e., oily, dry, and moist sites), the field is slowly moving towards understanding the role of the skin microbiota in sexually transmitted infections (STIs) [100]. One such example is the penile micro-environment, which possesses specific levels of oxygen availability, moisture content, and keratinization; these characteristics provide unique niches for penile microbiota to develop. However, information on the community structure of penile microbiota lags behind other skin sites, and initial studies mostly relied on culture-based approaches. Few studies have applied high-throughput sequencing approaches (16S rRNA sequencing) to characterize the penile microbiota, and results have shown that the human penile microbiota is composed of bacteria from the *Corynebacteriaceae*, *Pseudomonadaceae*, and *Oxalobacteraceae* families [101,102,103]. The diversity and composition of the penile microbiota can be influenced by practices such as circumcision [103], which is known to impact the predominance of opportunistic and pathogenic bacteria [104]. Recent interest in the penile microbiota stems from data indicating that circumcision reduces the risk of infections caused by human immunodeficiency virus (HIV) [105,106] and human papillomavirus (HPV) [107]. In the case of HIV, it is not yet clear whether specific penile microbiota increase the risk of infection or if HIV infection status influences the composition of penile microbiota [102]. Moreover, viral infections, such as those caused by HPV, seem to be more prevalent in men with a non-*Corynebacterium*-dominated penile microbiota [102]. Altogether, these results identify modulation of the skin microbiota as a means of preventing or treating disease.

### 5.3. Forensic Applications

The skin microbiota has been proposed as a tool for forensic applications due to its unique signatures, stability, and persistence. Skin bacteria can be unique to each individual and can often account for a high degree of interindividual variability [108], making skin bacteria potentially suitable for subject identification. In addition, the composition of skin bacteria is often stable over time and tends to return to its baseline, including after specific hygiene practices (e.g., hand washing) [109]. Skin bacteria can survive on surfaces and fomites for extended periods of time and persist in stressful conditions, including changes in moisture content and temperature or UV exposure [110]. A pioneering study assessing the suitability of the skin microbiota in forensics demonstrated that skin bacteria recovered from computer keyboards could be traced to their respective owner up to 2 weeks after last contact [111]. Other studies have utilized features of the *C. acnes* pangenome as a tool for skin microbiome profiling in forensic studies [112]. Another study found that certain skin bacteria can help distinguish individuals from different geographical regions, with *Cutibacterium* and *Streptococcus* frequently dominating the hands of individuals from the United States but not the hands of individuals from South Korea or Japan [113]. These studies demonstrate that the use of the skin microbiome in forensic applications will likely continue to evolve. In addition to using skin bacteria to identify individuals, objects, and time since contact, the skin microbiome has been proposed as a tool for estimating the time since death or the post-mortem interval (PMI) [114]. After demise, microbes take over the process of decomposition and putrefaction, a process affected by factors such as temperature, oxygen levels, surrounding soil [115,116], and the change in predictable successional processes [117].

In summary, as highlighted in the review by Zhang et. al., while many potential skin microbiome applications have been therapeutic in nature, there is evidence that it may also be an effective tool in diagnostics and forensics [117]. By studying the skin microbial community, it may be possible to assess the presence and state of acute (e.g., certain acne) and chronic skin conditions (e.g., atopic dermatitis and psoriasis) [118,119], as well as to connect individuals to geographies [113], identify individuals [111], measure the post-mortem interval [116], and even trace inter-individual contact through the transfer of skin microbiota [120]. These applications rely on identifying the unique microbial communities found on an individual, as well as understanding how active taxa associate with these various traits and conditions, including the process of decomposition.

### 5.4. Enabling ‘Omics Integration—Multi’omics

The application of various ‘omic technologies in skin microbiome research lags significantly behind other microbiome sites such as the gut microbiome, as highlighted by a number of studies. Some of these ‘omes, such as metatranscriptomics (described above), as well as metabolomics and metaproteomics, which quantify all the metabolites and proteins in a sample, respectively, have been mainly used in gut microbiome studies and have not been widely applied for skin microbiome research. This disconnect between gut and skin microbiome research in the application of various ‘omes, also known as multi’omics (Figure 2), may be partly due to the low-biomass nature of skin, as well as other factors mentioned above, which may affect downstream processes such as host depletion and sequencing depth. In turn, this can make skin microbiome studies using other ‘omes cost-prohibitive and time-consuming. Theoretically, the application of metabolomics and metaproteomics in skin microbiome research may enable the identification of metabolites and proteins associated with maintaining skin health or promoting discrete skin conditions. While a study applying human host metabolomics and transcriptomics analysis showed that a small fraction (10%) of the detectable metabolites and transcripts change during skin aging, determined by comparing young and aged epidermal tissue [121], there is a need to also understand the role of the skin microbiome’s metabolites and gene expression. As skin microbiome research shifts towards multi’omics approaches, a more holistic view of skin health and disease will emerge. In turn, this may enable the deciphering of multi-kingdom interactions and the development of novel and more effective therapeutics for dermal conditions. Notably, a multi’omics approach is not without limitations, as data normalization, integration, analysis and visualization require consideration of downstream objectives and analysis outcomes [122]. Therefore, it is anticipated that similar validation processes may be required for the integration of multiple ‘omes in skin microbiome research.

## 6. Concluding Summary

An effective study design and validated collection and extraction methods are critical first steps for low-biomass microbiome studies, such as those in skin. In addition, the choice of sequencing strategy used (i.e., untargeted metagenomic/metatranscriptomics sequencing vs. targeted 16S rRNA/ITS sequencing) is a critical decision to ensure success. Reference annotation can impact the interpretation of sequencing results for diagnostic and therapeutic objectives, and while the SILVA database is widely accepted in amplicon studies, no comprehensive shotgun metagenomics skin microbiome studies have evaluated the influence of database selection in taxonomic classification. Shotgun metagenomic and full-length 16S rRNA gene sequencing are increasingly being applied in skin microbiome studies to obtain species- and strain-level resolution. Metatranscriptomics is an emerging application for skin, and the technique holds potential to assess both taxonomy and the functional activity associated with the skin microbiota, as well as its interplay with the host. The addition of controls during sample collection, storage, extraction, amplification, sequencing, and analysis is essential for assessing biases and determining the bioburden and cross-contamination that may influence results and interpretation. As skin microbiome research moves forward, it is anticipated that technique validation will aid in furthering our understanding of the skin microbiota, provide a better understanding of multi-kingdom interactions, develop forensic applications, enable the integration of various ‘omics, and support the development of therapeutics for common skin diseases.

## Figures and Tables

**Table 1:** Important end-to-end considerations for a successful skin microbiome study. rRNA: ribosomal RNA.

Step	Key Considerations
Study design	Skin site and condition of interest (when applicable)
	Study power (i.e., number of participants and/or samples collected; relative abundance of signal(s) of interest)
	Participant metadata (e.g., ethnicity, age, biological sex, health status, use of medications, hygiene products, and/or cosmetics)
	Robust sampling procedure: area size vs. bioload, impact of hygiene, bioburden, etc.
	End-to-end review of the methods for compatibility and optimal sample performance
	Downstream analysis strategy compatibility
	Additional control(s): environmental/non-collected control
Sample collection/ storage	Means of sample collection (e.g., swab, scraping, biopsy, and tape-stripping) Validated and standardized for skin
	Low bioburden within device and contamination during collection
	Need for immediate freezing vs. inclusion of stabilization solution Storage length and conditions
Sample processing: nucleic acid extraction	Validated and standardized Optimized nucleic acid recovery
	Recovery of Gram-positive and Gram-negative bacteria and fungal species
	Effective clean-up of nucleic acids and removal of enzymatic inhibitors
	Low bioburden
	Extraction negative control
Sample processing: amplification and library preparation	Optimized for taxa (e.g., bacterial vs. fungal) of interest and biomass/host content Accurate capture of microbiome composition Optimal DNA input (for shotgun metagenomic and amplicon sequencing) and amplification conditions (for amplicon sequencing) Efficient removal of host and microbial rRNA and sufficient RNA input for metatranscriptomic sequencing
	Library preparation negative control
Bioinformatics: database selection	Updated and curated content source and data quality, removal or consolidation of redundant sequences, and comprehensiveness
	Level of taxonomic or functional resolution supported (e.g., genus, species, strain, and functional hierarchies)
	Suitability towards analysis strategy
Bioinformatics: annotation	Sensitivity and specificity of tool/approach Low false positive/false negative rate
	Database-dependent and database-independent approaches

**Table 2:** Examples of controls and environmental controls used in skin microbiome studies. This table also includes a description of the control, the main step(s) in which these controls are usually included, purpose(s), and expected outcome(s).

Control Type	Main Step(s) to Be Applied	Description	Purpose(s)	Expected Outcome(s)
Extraction blank	Nucleic acid extraction/ library preparation	Type of negative control containing no sample material that is processed in parallel with sample(s) of interest during the extraction process.	Assess the kitome and the introduction of environmental contaminants or cross-contamination; analytic assessment of sample similarity to environment/reagents.	Negligible nucleic acid concentrations; no/little amplification during library preparation; low read counts; significantly differentiated from sample in analysis.
Negative control (amplification)	Library preparation	Type of negative control included during sample preparation that is expected to produce no library.	Assess contamination introduced during library preparation.	No/little amplification during library preparation.
Positive control (amplification)	Library preparation	Type of positive control included during sample preparation.	Ensures that library preparation was successful.	Library of the expected size and yield.
Mock community		Type of control composed of a defined mixture and composition of cells/viruses, nucleic acids, or in silico genomes.	Assess process efficiency, accuracy, and sensitivity from nucleic acid extraction to data analysis.	The identification of expected organisms, the measurement of their proportions, and a comparison to the expected/ground-truth, measurements of sensitivity and specificity (false positive/false negative rates).
Cells/Viruses	Nucleic acid extraction/ library preparation/ database selection/ annotation	Single or multiple collection of cells (e.g., bacterial and fungal) or viruses relevant for study objectives.	Assess nucleic acid extraction, library preparation, sequencing, and analysis efficiency.	End-to-end assay compatibility with taxonomic target(s) (e.g., for the development of a diagnostic measurement).
Nucleic acids	Library preparation/ database selection/ annotation	Type of mock community composed of nucleic acids (DNA).	Assess library preparation, sequencing, and analysis efficiency	
In silico genomes	Database selection/ annotation	Type of mock community composed of in silico reference genomes/known sequences.	Assess analysis efficiency and accuracy.	

## Data Availability

Not applicable.
